# Knowledge, attitude, and practices of restaurant and foodservice personnel in food allergy. A systematic review and meta-analysis

**DOI:** 10.1016/j.heliyon.2024.e33431

**Published:** 2024-06-24

**Authors:** Ximena A. Figueroa-Gómez, María Jesús Oliveras-López, Marcelo F. Poyanco-Bugueño, Francisco M. Ocaña-Peinado, Herminia López-García de la Serrana, Magdalena Araya Quezada

**Affiliations:** a Nutrition and Food Science Doctoral Program at the Department of Nutrition and Bromatology, Faculty of Pharmacy, University of Granada, 18071, Granada, Spain; b Nutrition and Food Science Doctoral Program at the Human Nutrition Unit, Institute of Nutrition and Food Technology (INTA), University of Chile, El Líbano 5524, CP. 7830490, Macul, Santiago, Chile; c Department of Molecular Biology and Biochemical Engineering, University Pablo de Olavide, Carretera de Utrera, Km 1, 41013, Seville, Spain; d University of Valparaíso, Blanco 951, Valparaíso, Chile; e Department of Statistics and Operations Research, University of Granada, 18071, Granada, Spain; f Department of Nutrition and Bromatology, Faculty of Pharmacy, University of Granada, 18071, Granada, Spain; g Institute of Nutrition and Food Technology (INTA), University of Chile, El Líbano 5524, CP. 7830490, Macul, Santiago, Chile

**Keywords:** Food allergy, Food service, Restaurants, Knowledge, Attitude, Practices, Food service staff

## Abstract

**Background:**

Currently, there may be 240–250 million people worldwide affected by food allergies. Dining out can be challenging for individuals with food allergies who rely on restaurant and food service staff to properly prepare allergen-free meals. For this reason, the personnel working in restaurants and other food services play a significant role in managing the risks faced by customers with food allergies.

**Objectives:**

A systematic review and meta-analysis was conducted to assess the existing evidence concerning the knowledge, attitudes, and practices related to food allergies among restaurant and foodservice personnel.

**Methods:**

To identify, characterize, and synthesize published research on the prevalence of positive responses regarding knowledge, attitudes, and practices related to food allergies among restaurant and food service personnel, international recommendations for systematic reviews and PRISMA guidelines were followed. The search was conducted between January 2012 and January 2022, utilizing the electronic databases PubMed, Web of Science, Scopus, and the Cochrane Library. Selection and data extraction were carried out following predefined protocols, and constructs based on reported outcomes were generated and subsequently analyzed in the meta-analysis. Trials were evaluated using the Cochrane tool for risk of bias. The results are presented using summary tables, forest plots, and box plots, showcasing the combined proportion of constructs obtained from independent surveys conducted without control groups. These constructs were then grouped into categories as an organizational framework and analyzed to determine their distribution among quintiles, aiming to provide a detailed overview of data variability. This strategy allowed us to demonstrate how results from the analyzed categories were distributed.

**Results:**

The algorithm identified 23 relevant studies, primarily originating from the United States and Europe. The main observed variability was related to the evaluated populations and the instruments used. A total of 28 constructs were identified, with 10 related to knowledge, 9 to attitudes, and 9 to practices. A proportion meta-analysis was conducted to determine the prevalence of positive responses within these three study categories. The results obtained reveal that, in the knowledge category, quintiles 4 and 5 exhibit strong knowledge on the subject (over 84 %). In contrast, starting from quintile 2, the majority of participants shows a positive attitude toward catering to consumers with food allergies (over 85 %). However, after examining the practices category, responses belonging to quintile 5 reflect a low level of risk, while in the other quintiles, behaviors with a higher potential risk for consumers with food allergies are identified.

**Conclusions:**

The knowledge, attitudes, and practices of personnel in the food service sector are crucial due to the increasing prevalence of food allergies today, as well as the growing frequency of eating out. Knowledge is the most extensively studied category, showing generally acceptable but still insufficient levels in some areas. Positive attitudes are expressed towards individuals disclosing allergic conditions, yet they do not necessarily correlate with high levels of knowledge. The identified practices do not ensure the safety of the dish served to the customer. There is limited awareness regarding the importance of preventing acute allergic reactions at the time of food consumption. Restaurants and food services should train all staff involved in customer service, implement protocols aimed at preventing allergic reactions during food service, and establish guidelines for handling a customer experiencing an acute reaction.

The limitations of this research are related to the heterogeneity present in the synthesized results, urging caution when interpreting the overall estimate of the combined effect, as the findings may not be applicable to all populations or study settings. Indeed, more studies are needed to enhance result precision and provide more specific recommendations for catering to allergic customers in restaurants and food services.

## 1 Introduction

Food allergy (FA) involves an immune-mediated adverse reaction triggered by food components, known as allergens, which are primarily proteins [1]. These reactions encompass one of three types of immune responses: IgE-mediated (rapid, potentially life-threatening symptoms), non-IgE-mediated (symptoms appearing hours to days after exposure), and mixed (both mechanisms involved) [2].

Similar to other immune-mediated diseases, the prevalence of FA is increasing globally. Currently, 32 million people in the U.S [3]. and between 11 and 26 million in Europe are affected. When extrapolated globally, these figures suggest a potential 240–250 million people affected, clearly signifying a substantial disease burden. Available evidence indicates that young children develop more FA (5–8%) than adults (1–2%) [4]. The reasons for the global rise remain unclear; accepted hypotheses often point to environmental factors, such as the dramatically changing lifestyles and dietary habits that occurred during the past century [5].

Regarding food triggers for FA, over 170 identified foods can incite adverse immune reactions. However, most countries list a shorter set of the most common local allergens leading to FA and regulate their use. For example, the U.S. identifies 9 allergens (milk, egg, peanuts, tree nuts, wheat, soy, fish, shellfish, and sesame) [6] while Europe lists 14, including celery, gluten-containing cereals (like barley and oats), shellfish (such as shrimp, crabs, and lobsters), eggs, fish, lupine, milk, mollusks (such as mussels and oysters), mustard, peanuts, sesame, soy, sulfur dioxide, and sulfites.

To date, removing the causal agent from the diet remains the only effective treatment for FA [7]. For allergic patients, eating out becomes a significant challenge, as restaurants and public food venues often do not guarantee that the served food is safe, i.e., that the allergens to be avoided are genuinely absent. Avoiding a specific allergen when preparing food typically means avoiding it as an ingredient, often ignoring its presence in additives or due to cross-contamination [8]. Nearly half of reported fatal allergic reactions over a 13-year study were associated with dining in restaurants or food services [9]. A U.S. national registry reported that 19 % of food-related deaths occurred in restaurants [10,11]. According to WHO, food establishment owners must ensure the safety of the food they offer, as both they and the service personnel are responsible for processing, transporting, and handling food safely. Simultaneously, consumers require timely information about the risks associated with their condition(s) and nutritional status [12].

When consumers disclose their allergies, the service staff must provide information about the menu, ingredients used, potential added allergens, and possible cross-contamination [13]. The fact that personnel might be unaware of current regulations regarding allergens underscores the need for education and training on allergic conditions, health risks, and optimal food safety practices, including the implementation of quality control programs such as Hazard Analysis and Critical Control Points (HACCP) [14,15]. This is crucial for improving the current situation, which is often precarious in many countries. Studies show that employees working in restaurants and other food services often have not received training about the implications of FAs and their dietary requirements [16,17], hindering understanding and appropriate responses to questions and needs of allergic consumers [17]. Today, there is widespread consensus that restaurants and food service managers should be knowledgeable about food allergies and regulations, and how this translates into food service operations [18].

Although we have conducted a comprehensive analysis of the available scientific literature to date, we have not conclusively identified any previous reviews that directly address the topic of our study, except for a prior study [19] that investigated the prevalence of correct responses regarding knowledge and practices of restaurant staff in relation to food allergies and celiac disease through a systematic review and meta-analysis. However, this study mainly focused on the training received by these individuals and the knowledge acquired after training. In our systematic review, we wanted to focus not only on knowledge, but also on the attitude and practices that food service personnel have regarding the care of customers who have special dietary needs due to food allergies.

## 2 Materials and methods

This systematic review adhered to international recommendations for systematic reviews [20,21], and a copy of the PRISMA 2020 checklist [22] is available in S1 File.

### 2.1 Protocol and registration

Registration of this protocol in PROSPERO was not required, as the platform is not intended for reviews primarily focused on food control and management research topics.

### 2.2 Eligibility criteria

The eligibility criteria for this study were defined using the PICO approach. The research question was, “What is the prevalence of positive responses in studies analyzing the knowledge, attitudes, and practices of restaurant and food service personnel regarding food allergies or celiac disease?” (S2 File - 1).

### 2.3 Inclusion criteria

The population of interest included restaurant or foodservice personnel, including managers, chefs, cooks, waitstaff, kitchen assistants, etc. We opted for broad inclusion covering all restaurant or food service staff, from executives to assistants. This allowed us to capture a comprehensive view of perceptions regarding food allergies and dietary needs without focusing on specific differences between professional roles. Scientific articles with quantitative studies that assessed knowledge about food allergies, attitudes toward customers with special dietary needs, and practices in food handling by restaurant and foodservice personnel were included. Articles published within the last decade were included, as studies older than this time frame may be considered outdated. Articles written in any language were accepted.

### 2.4 Exclusion criteria

The following exclusion criteria were applied [1]: Book sections, patents, reviews, letters, conference abstracts, case reports, brief communications, and thesis chapters [2]; Systematic reviews [3]; Studies containing only qualitative research [4]; Research focused on personnel from educational institutions such as daycares, schools, or universities; and [5] Healthcare institutions such as clinics or hospitals.

### 2.5 Sources of information and search strategies

The search was conducted from January 2012 to January 2022 in the electronic databases of PubMed, Web of Science, Scopus, and the Cochrane Library.

Initially, a partial bibliographic search was performed on Google Scholar, which yielded some relevant articles from which essential keywords for the research were extracted. Different combinations were tested using these keywords, and an optimal equation was obtained as follows:

((“food hypersensitivity” OR “food allergy” OR “celiac disease” OR “gluten free”) AND ((“food services” OR “eating out” OR “restaurants” OR “hotel” OR “fast food” OR “kitchen”) OR (“chefs” OR “manager” OR “cooks” OR “waiter” OR “kitchen workers” OR “staff” OR “food handlers")) AND ((training) OR (control OR management OR treatment) OR (awareness OR behaviours OR experience OR knowledge OR practice)) NOT immunotherapy).

### 2.6 Selection process

As a result of the database search, a total of 1126 articles were retrieved, which were managed using the Mendeley and subsequently Zotero desktop programs. After removing duplicates and unavailable articles, i.e., those not accessible online or due to subscription limitations, the references were exported to a spreadsheet (Excel, Microsoft Office LTSC Standard 2021) to facilitate the review process. One additional duplicate was manually identified and removed, and 13 references that were not primary quantitative research studies (such as patents, book sections, communications, etc.) were excluded. This left 884 articles for analysis. During this process, the relevance of titles and abstracts of the identified studies from the search was assessed using the guiding question: Is this a primary quantitative research study investigating the knowledge, attitudes, and/or practices of restaurant and foodservice personnel regarding food allergies and celiac disease? Subsequently, Reviewers 1 and 2 analyzed the remaining 37 articles, eliminating those that did not meet the eligibility criteria. Based on the abstract, 8 articles were excluded for not meeting research criteria. Finally, 9 more articles were excluded after reading the full text due to inadequate data in their results section.

Throughout the process, in cases of disagreement, the issue was discussed until consensus was reached between the reviewers. When consensus couldn't be achieved, a third reviewer (Reviewer 3) made the final decision. The final selection was always based on the full text of the publication. The list of references from the selected studies was critically evaluated by Reviewer 4. Ultimately, 20 relevant articles were accepted for evaluation.

To verify the selection, a manual search was conducted in the references of all relevant articles, adding three additional studies that were not detected in the electronic database search. Thus, a total of 23 scientific articles were selected for the study.

### 2.7 Data collection process

Subsequently, a systematic review form proposed by Young [19](S2 File - 2) was employed to assess article relevance, confirmation, and characterization. This form was adapted to suit the specific needs of this study, optimizing both its size and question quality. Accomplishing this was achieved by reducing and adjusting the questions, ensuring a concise and efficient data collection process for this investigation.

Articles were categorized according to their features (publication year, geographic location, analytical design, research focus, target population, etc.). Disagreements among reviewers were resolved through discussion, with the third author (Author 3) arbitrating unresolved disputes. The outcomes of the form's categorization application are available in S3 File.

### 2.8 Evaluation of risk of bias

In the assessment of bias in the selected studies, the reviewers (Reviewer 1 and Reviewer 2) applied standard questions from the Cochrane Handbook for Systematic Reviews of Interventions 5.1.0. This methodology, endorsed by Cochrane, ensures consistency and credibility in assessments for both clinical and non-clinical studies. Additionally, these versatile tools enhance the methodological robustness of our analysis, guaranteeing the reliability of our results. The following criteria were applied [1]: selection bias [2], performance bias [3], detection bias [4], attrition bias [5], reporting bias, and [6] other biases for the 23 studies that contributed to the 28 constructs across the three areas of research. The risk of bias was categorized as “high,” “low,” or “unclear” for each criterion. Reviewers [[1], [2]], independently and in duplicate, were responsible for assessing the risk of bias, with the assistance of Reviewer [3]. The risk of bias assessment is available in Supplementary File 5.

### 2.9 Generation of constructs

During the construct generation phase, selected articles were characterized, and the questionnaires used to assess restaurant staff in the 23 included studies were reviewed. Questions with identical or similar content were extracted from these questionnaires and grouped to form representative constructs for analysis. The inclusion of at least three studies was used as a criterion for accepting a construct.

Once a construct was identified, employee responses were analyzed using specific criteria. Criteria based on alignment with the scientific consensus on food allergy were used to assess knowledge. In the attitude category, a proactive and positive attitude toward the safety and well-being of allergic customers was sought, along with a willingness to learn and improve skills in managing these needs. In the Practices category, the evaluation went beyond theoretical knowledge to include staff behavior in effectively implementing safe and responsible practices for customers with food allergies. These rigorous criteria allowed us to measure the prevalence of each construct accurately and consistently.

It is worth noting that in cases where studies did not provide the exact number of responses, estimates were made based on the percentages provided. The identified constructs were then categorized into three groups: Knowledge, Attitudes, and Practices. Additionally, alongside assessing theoretical knowledge, the attitudes and practices categories included actual staff behavior in managing the needs of allergic clients. A total of 10 constructs were developed to assess correct responses in the Knowledge category, 9 for the Attitudes category, and another 9 for the Practices category related to food allergies. Supplementary file S4 contains further details on these categories and constructs.

## 3 Meta-analysis and synthesis

### 3.1 Meta-analysis

In this stage of the research, we meticulously analyzed 28 aspects related to the knowledge, attitudes, and practices of food service staff regarding food hypersensitivity. The studies were carefully selected from accredited databases, following strict inclusion criteria to ensure the relevance and quality of the collected data. The obtained results were categorized into three distinct areas: knowledge, where correct responses regarding allergen information were assessed; positive attitudes towards customers with food hypersensitivity; and low-risk practices in collective catering service environments.

For data analysis, we utilized Microsoft Excel and calculated 95 % confidence intervals of the combined proportion using a logarithmic transformation. This technique enhanced the accuracy of variability estimates. The 95 % confidence intervals for the combined proportion were calculated using a logarithmic transformation of the data, enabling us to estimate variability precisely and provide confidence limits for the combined proportion.

Regarding the measurement of the effect size for each construct, we utilized the weighted mean method known as Cohen's d. This method enabled us to compute the individual effect size for each included study in the meta-analysis, resulting in an overall measurement of the effect size for each construct. When performing individual meta-analysis for each construct, we assessed the incidence of positive responses about food allergies gathered from questionnaires in studies that contributed to each construct. To combine data from multiple studies, we applied the Mantel-Haenszel pooled proportions method [23]. This statistical technique enabled us to calculate an aggregated estimate of the prevalence of positive responses in the analyzed population without the need for a control group in each study.

We conducted a descriptive analysis of the data derived from the selected studies, assessing the heterogeneity of constructs to determine their potential influence on the precision and validity of resulting estimates. Additionally, summary measures such as the median, 25th percentile, and 75th percentile were applied to synthesize the distribution of prevalence estimates for each construct. The visual presentation of these results was done through Forest plots [24] and box and whisker plots, using Microsoft Excel, Microsoft Office LTSC Standard 2021 version.

### 3.2 Synthesis of categories

Following individual meta-analyses, we grouped the results into three main categories: knowledge, attitudes, and practices. This practice facilitated a structured understanding of the results and their subsequent interpretation. The data were organized into quintiles to clearly display the variability in positive responses, providing an effective understanding of how responses were distributed in the research. These results can be found in Supplementary File 7 (S7).

### 3.3 Evaluation of reporting bias, validity, and precision

This investigation utilized rigorous measures to mitigate information bias. The study adhered to international protocols for systematic reviews, including a comprehensive literature search across multiple databases. Furthermore, critical assessments of the included studies were conducted to identify potential information bias and other forms of bias. Clear and transparent criteria were utilized for study selection. Heterogeneity was also measured for each construct. The study did not explore the causes of such heterogeneity, as the primary purpose was to provide a preliminary synthesis of the topics under investigation. Collectively, these steps served to diminish the risk of information bias and enhance the validity and precision of the systematic review and meta-analysis findings.

Moreover, a 95 % confidence interval was calculated for each study, and a detailed assessment of the risk of bias was performed for each construct. The outcomes are depicted in Forest plot graphs, aiding in the visualization of result variability.

## 4 Results

**Fig. 1:**
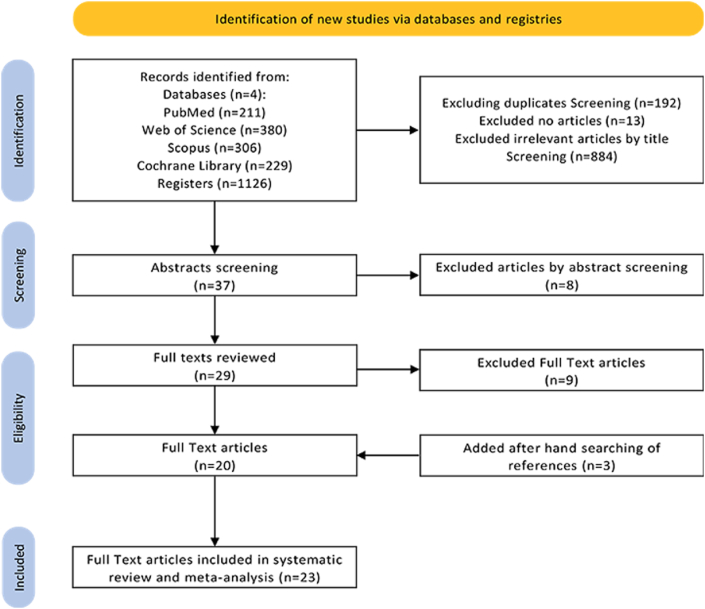
Prisma flow diagram for the systematic review.

The applied search algorithm identified 1126 articles, which, after removing duplicates, non-scientific articles, and articles not relevant based on title, left 37 studies. Eight additional articles were excluded after reviewing their abstracts; in five cases, full texts were not available, and in four cases, the reported results were incomplete. As a result, 20 relevant articles remained for final analysis. After a thorough review of the complete articles and their references, three relevant articles that were not previously detected in electronic databases (one of them published in 2011) were added, resulting in a final set of 23 articles for evaluation. No articles were found that specifically examined the knowledge, attitudes, or practices of personnel regarding food intolerances and celiac disease. Therefore, our research focused exclusively on food allergies. (Fig. 1).

### 4.1 Characterization of selected studies

Among the 23 articles published between 2011 and 2022, 34.8 % (n = 8) came from the United States, 39.1 % (n = 9) from Europe (3 from the United Kingdom and 2 from Turkey). The majority of the studies (n = 21) adopted a cross-sectional design, while two employed an experimental design.

In all the studies, data collection was carried out through questionnaires, although only 34.8 % (n = 8) confirmed having subjected them to a pre-test before their application. In 43.5 % (n = 10) of the cases, participants took part in face-to-face interviews, while the rest (56.5 %, n = 13) were approached using various methods such as email, phone calls, and surveys conducted by specialized companies.

Out of the 23 studies, 22 focused on restaurants and one on restaurants in Turkish tourist hotels [25].

The selected studies had a diverse target population: owners accounted for 34.8 % (n = 8 studies), managers for 73.9 % (n = 17 studies), and supervisors for 8.7 % (n = 2 studies). Additionally, chefs represented 43.5 % (n = 10 studies), cooks 60.9 % (n = 14 studies), and waiters 60.9 % (n = 14 studies), among others (refer to Table 1).

**Table 1:** General characteristics of the 23 selected articles.

**General characteristics**	**Frequency (n)**	**Percentage (%)**
**Country of origin**		
**North America**		
United States of America	8	34,8
Canada	1	4,3
**Middle Orient**		
Turkey	2	8,7
Lebanon	1	4,3
**Europe**		
United Kingdom	3	13,0
Spain	1	4,3
Romania	1	4,3
Germany	1	4,3
Latvia	1	4,3
Poland	1	4,3
Portugal	1	4,3
**Asia**		
Malaysia	1	4,3
**Oceania**		
New Zealand	1	4,3
**Study design**		
Cross-sectional study	20	86,9
Experimental	3	13,1
**Type of food services included:**		
Restaurants	22	95,7
Cafes	4	0,92
Fast food	2	0,46
Restaurants at hotels	1	0,23
Catering	1	^b^,23
Take away	2	0,46
**Type of personnel included**^a^, ^b^:		
Category of owners, managers or supervisors	18	78,3
Category of chefs, cooks or kitchen^a^assistants	17	73,9
Waiter category receptionist/cashier or hosts	1^a^	60,9
Not specified	5	21,7
**Outcomes**^a^		
Knowledge	23	100
Attitudes	13	56,5
Practices	11	47,8
Knowledge, attitudes and practices all together	7	30,4

a Multiple selections were possible for these questions, and percentages may not add up to 100 %.

b studies that interviewed at least one of the persons study categories.

All of the studies evaluated knowledge related to food allergies. 56.5 % (n = 13) investigated the staff's attitudes, and 47.8 % (n = 11) examined practices associated with the management of allergenic foods in restaurants. Seven studies assessed the knowledge, attitude, and practices of the staff simultaneously.

For an overview of the characteristics of the 23 investigated articles, refer to Table 2.

**Table 2:** A summary of 23 articles included in the analysis that investigated Knowledge, attitude, and practices of restaurant and foodservice personnel in food allergy.

**Author**	**Country**	**References**	**Knowledge**	**Attitude**	**Practices**
Bailey et al. (2011)	United Kingdom	Restaurant staff's knowledge of anaphylaxis and dietary care of people with allergies	**✓**		
Wojtyniak et al. (2013)	Poland	Knowledge of food allergy among staff of Warsaw restaurants	**✓**		
Bailey et al. (2014)	United Kingdom	Food allergy training event for restaurant staff; A pilot evaluation	**✓**	**✓**	
Wham and Sharma (2014)	New Zealand	Knowledge of café and restaurant managers to provide a safe meal to food allergic consumers	**✓**		
Sogut et al. (2015)	Turkey	Food allergy knowledge and attitude of restaurant personnel in Turkey	**✓**	**✓**	
Shafie and Azman (2015)	Malaysia	Assessment of knowledge, attitude and practice of food allergies among food handlers in the state of Penang, Malaysia.	**✓**	**✓**	**✓**
Lee and Xu (2015)	USA	Food Allergy Knowledge, Attitudes, and Preparedness Among Restaurant Managerial Staff	**✓**	**✓**	**✓**
Radke et al. (2016)	USA	Food allergy knowledge and attitudes of restaurant managers and staff: An EHS-Net study	**✓**	**✓**	
Lee and Sozen (2016)	USA	Food allergy knowledge and training among restaurant employees	**✓**		
Dupuis et al. (2016)	USA	Food allergy management among restaurant workers in a large U.S. city	**✓**		**✓**
Lessa et al. (2016)	Spain	Food Allergy Knowledge, Attitudes and Practices: A Pilot Study of the General Public and Food Handlers	**✓**	**✓**	**✓**
Radke et al. (2017)	USA	Restaurant food allergy practices - six selected sites, United States, 2014	**✓**		**✓**
Wen and Kwon (2017)	USA	Restaurant servers' risk perceptions and risk communication-related behaviors when serving customers with food allergies in the U. S	**✓**	**✓**	
Lee and Barker (2017)	USA	Comparison of Food Allergy Policies and Training between Alabama (AL) and National Restaurant Industry	**✓**		
Lee and Sozen (2018)	USA	Who knows more about food allergies - restaurant managerial staff or employees?	**✓**	**✓**	
McAdams et al. (2018)	Canada	Food allergy knowledge, attitudes, and resources of restaurant employees	**✓**	**✓**	**✓**
Soon (2018)	United Kingdom	‘No nuts please': Food allergen management in takeaways	**✓**	**✓**	**✓**
Jianu and Golet (2019)	Romania	Food Allergies: Knowledge and Practice among Food Service Workers Operating in Western Romania	**✓**		**✓**
Loerbroks et al. (2019)	Germany	Food allergy knowledge, attitudes and their determinants among restaurant staff: A cross-sectional study	**✓**	**✓**	
Bujaka and Riekstina-Dolge (2019)	Latvia	Food allergy knowledge and practice of restaurant staff	**✓**		**✓**
Pádua et al. (2020)	Portugal	Impact of a web-based program to improve food allergy management in schools and restaurants	**✓**		
Nasseredine et al. (2021)	Lebanon	Food allergy knowledge, attitudes and practices of foodservice workers at restaurants in Lebanon: Findings from a national cross-sectional study	**✓**	**✓**	**✓**
Eren et al. (2021)	Turkey	Food allergy knowledge, attitude, and practices of chefs in resort hotels in Turkey	**✓**	**✓**	**✓**
Total			n = 23 (100 %)	n = 13 (56,5 %)	n = 11 (47,8 %)

### 4.2 Bias risk

Among the six criteria scrutinized for bias analysis, 81 % of the articles were categorized as having a low risk of bias. Conversely, 18.9 % were considered as “risk not clear” in at least one of the evaluated criteria. Across the 23 examined studies, there were a total of 110 knowledge assessments, 40 attitude assessments, and 46 practice assessments, culminating in 196 risk assessments. Among these 196 evaluations (as shown in Table 3), only one construct (related to knowledge) was identified as having a “high risk” of bias.

**Table 3:** Bias risk in 23 studies that evaluated food allergy related knowledge, attitudes and practices in personnel that works in restaurants and other food services.

Category	Evaluations (n)	Classification of bias risk n (%)		
		Low risk	Unclear risk	High risk
Selection	196	102 (52,0 %)	93 (47,4 %)	1 (0,5 %)
Performance	196	156 (79,6 %)	40 (20,4 %)	0 (0,0 %)
Detection	196	171 (87,2 %)	24 (12,2 %)	1 (0,5 %)
Attrition	196	141 (71,9 %)	55 (28,1 %)	0 (0,0 %)
Reporting	196	186 (94,9 %)	10 (5,1 %)	0 (0,0 %)
Other biases	196	196 (100,0 %)	0 (0,0 %)	0 (0,0 %)

Definitions of risk: low risk = plausible risk with low probability of significantly changing the results; unclear risk = plausible risk that makes results to be doubtful. High risk = plausible risk that makes results considerably weak.

Identification and Analysis of the Knowledge Category.

Within the category knowledge of food service personnel regarding food allergies, a total of 10 constructs were identified (Table 4). Complete details of these analyses are shown in File S6.

**Table 4:** 28 constructs generated: 10 knowledge, 9 attitudes and 9 practices of staff working in restaurants and other food services in relation to food allergies.

**Category**	**Id**	**Constructs**
**Knowledge (K)**	K1	Customers with food allergies can safely consume small portions of the specific food thy are allergic to (False)
	K2	If an individual is having an allergic reaction, serving them water will dilute the allergen and relieve the reaction (False)
	K3	High heat (e.g. frying in hot oil) destroys the majority og food allergens (False)
	K4	A food allergic person can die from eating the food that is allergic to (True)
	K5	Removing the allergen from an already ready-to-serve dish makes it safe for the person allergic to it (False)
	K6	Lactose intolerance and milk allergy are the same thing (False)
	K7	The most effective management for a severe food allergy reaction is administering epinephrine (True)
	K8	Modern medicine can cure food allergies. (False)
	K9	If the buffet in a help-yourself service contains allergens, to keep it clean is enough to make it a safe option for food allergic customers (False)
	K10	A food allergic reaction can occur if a client touches a food item that contains the allergens, he/she is allergic to. (True).
**Attitude (A)**	A1	Kitchen staff should be aware of food allergies.
	A2	Restaurants should try to satisfy special requests made by customers with food allergies.
	A3	Do you think that food allergies are a serious issue worth consideration?
	A4	I believe I can handle correctly an emergency food allergy situation at my workplace
	A5	Have you ever thought how to prevent food allergy reactions among your customers?
	A6	Do you think that you are responsible for the presence of food allergens in your allergic customer served foods?
	A7	It is customers’ responsibility to express their food allergies needs.
	A8	I know that I can provide a safe meal to clients that inform their special needs.
	A9	Would you like to receive further education on food allergies?
**Practice (P)**	P1	Do you have a plan to provide safe meals to allergic clients?
	P2	Would you modify a recipe for food allergy customers who request it?
	P3	On your menu, do you highlight allergenic ingredients or insert a warning note to inform the presence of specific allergens like peanuts or others?
	P4	I wash my hands with soap and water and change my gloves before processing allergen-free foods
	P5	I post information about food allergies in the restaurant's website
	P6	Sometimes I fry allergen-free foods in the same oil where we previously fried allergen containing foods
	P7	Does this restaurant have a special set of utensils or equipment to prepare allergen-free food?
	P8	Does this restaurant have a list of procedures with the menu recipes indicating the ingredients they are made of?
	P9	Do the personnel have training focused on how to receive and deal with consumers that inform food allergies?

**Fig. 2:**
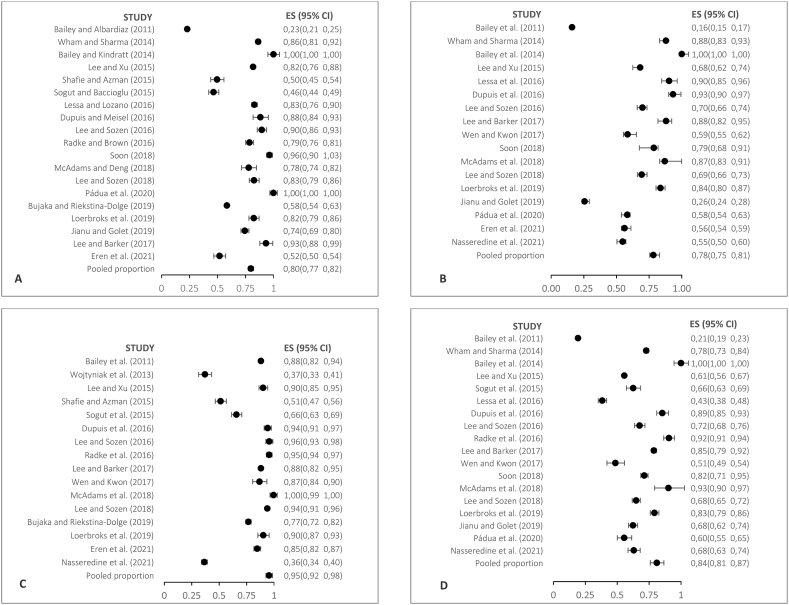
Forest plot of representative examples of prevalence of knowledge observed in personnel that works in restaurants and other food services. (A) K1. Customers with food allergies can safely consume small portions of the specific food they are allergic to (False) (B) K3. High heat (e.g., frying in hot oil) destroys the majority og food allergens (False) (C) K4. A food allergic person can die from eating the food that is allergic to (True) (D) K5. Removing the allergen from an already ready-to-serve dish makes it safe for the person allergic to it (False).

Fig. 2 shows the highlighted results in this category, as they involve a larger number of studies.

For instance, in construct K1, evaluated through true or false statements, 79.8 % (n = 3815) of the surveyed individuals across 19 studies correctly responded that it is false that customers with food allergies can safely consume small portions of the allergen-containing food.

Within construct K3, the notion that high heat, as in frying in hot oil, destroys most food allergens was explored. The results demonstrated that 78.3 % (n = 3002) of food service personnel surveyed in a total of 17 studies correctly confirmed this statement to be false.

In the context of construct K4, the belief that a person allergic to a food could die from consuming the specific allergen was addressed. In this case, virtually all respondents (95.4 %, n = 3844) across 16 studies acknowledged that ingesting an allergen can potentially be lethal for someone with a food allergy.

Lastly, in construct K5, concerning food preparation, the (wrong) idea of whether removing the allergen from a ready-to-serve dish would make it safe for an allergic individual was investigated. About 83.8 % (n = 3481) of respondents across 18 studies believed that the food would still be unsafe if the allergen is removed directly from the ready-to-serve dish.

### 4.3 Identification and analysis of the attitude category

**Fig. 3:**
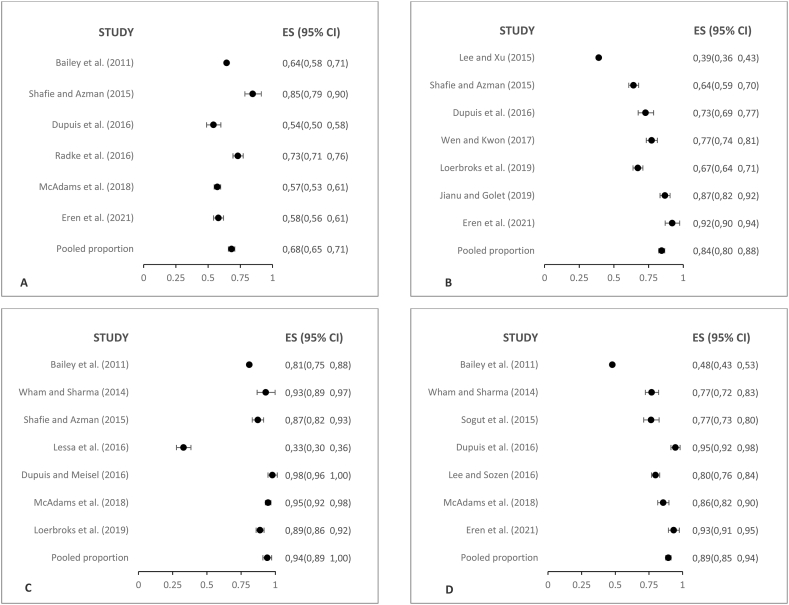
Forest plot of representative examples of prevalence of attitudes observed in personnel that works in restaurants and other food services. (A) A4. I believe I can handle correctly an emergency food allergy situation at my workplace (B) A6. Do you think that you are responsible for the presence of food allergens in your allergic customer served foods? (C) A8. I know that I can provide a safe meal to clients that inform their special needs. (D) A9. Would you like to receive further education on food allergies.?.

Within the category addressing the attitudes of food service personnel regarding food allergies, a total of 9 constructs have been identified (Table 4). Complete details of this analysis can be found in File S6. Fig. 3 shows the highlighted results in this category, as they involve a larger number of studies.

Construct A4 focuses on the perception of being able to handle emergency situations related to food allergies in the workplace; 68.2 % (n = 1752) of the surveyed personnel across 6 studies expressed confidence in their ability to properly manage such critical situations.

Within the context of construct A6, the perspective on the responsibility of ensuring the absence of food allergens in dishes served to allergic customers was explored. A notable 84.4 % (n = 1726) of responders in the 7 surveyed studies believed that ensuring food safety for each customer with a food allergy is their personal duty.

In construct A8, the perception about the ability to provide safe meals to customers who report special dietary needs was addressed. The majority of respondents (an impressive 94.1 %, n = 1101) across seven studies expressed confidence in their training to offer safe food to individuals with food allergies.

Lastly, in construction A9, regarding the interest in acquiring more information about food allergies, 89.4 % (n = 1703) of the surveyed staff in the 7 studies assessed expressed their eagerness to learn more about this topic.

### 4.4 Identification and analysis of the practice category

**Fig. .4:**
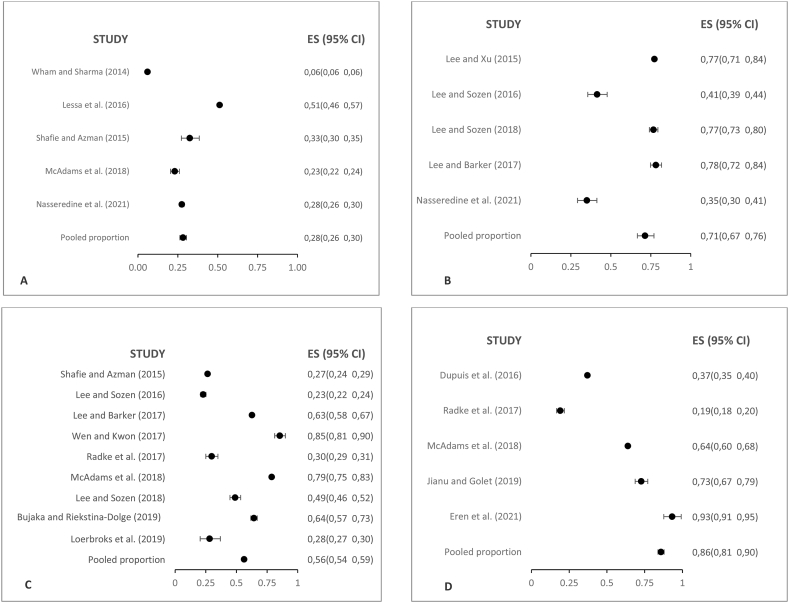
Forest plot of representative examples of prevalence of practices observed in personnel that works in restaurants and other food services. (A) P1. Do you have a plan to provide safe meals to allergic clients? (B) P2. Would you modify a recipe for food allergy customers who request it? (C) P3. On your menu, do you highlight allergenic ingredients or insert a warning note to inform the presence of specific allergens like peanuts or others? (D) P7. Does this restaurant have a special set of utensils or equipment to prepare allergen-free food.?.

Within the category addressing the practice of food service personnel regarding food allergies, a total of 9 constructs have been identified (Table 4). Complete details of this analysis can be found in File S6. Fig. 4 shows the highlighted results in this category, as they involve a larger number of studies.

In construct P1, related to the existence of a plan to provide safe meals to allergic customers, only 28.3 % (n = 666) of the surveyed personnel across 5 studies indicated the implementation of secure protocols to meet the special dietary needs of customers.

Within the context of construct P2, which addresses the willingness to modify recipes for customers with food allergies, a significant 71.4 % (n = 828) of the surveyed personnel across 5 studies expressed their readiness to make modifications to culinary preparations according to each customer's specific needs.

In construct P3, addressing the visibility of allergenic ingredients on the menus as well as the inclusion of warnings about the presence of specific allergens, almost half of the participants (56.3 %, n = 2215) across 9 studies confirmed that they indeed highlight allergen information on the food service menu where they work.

In construct P7, regarding the availability of special tools and equipment to prevent cross-contamination, a significant 63.9 % (n = 1307) of the surveyed staff across five studies reported using specific tools and equipment for preparing food intended for individuals with allergies, therefore ensuring improved safety.

**Fig. 5:**
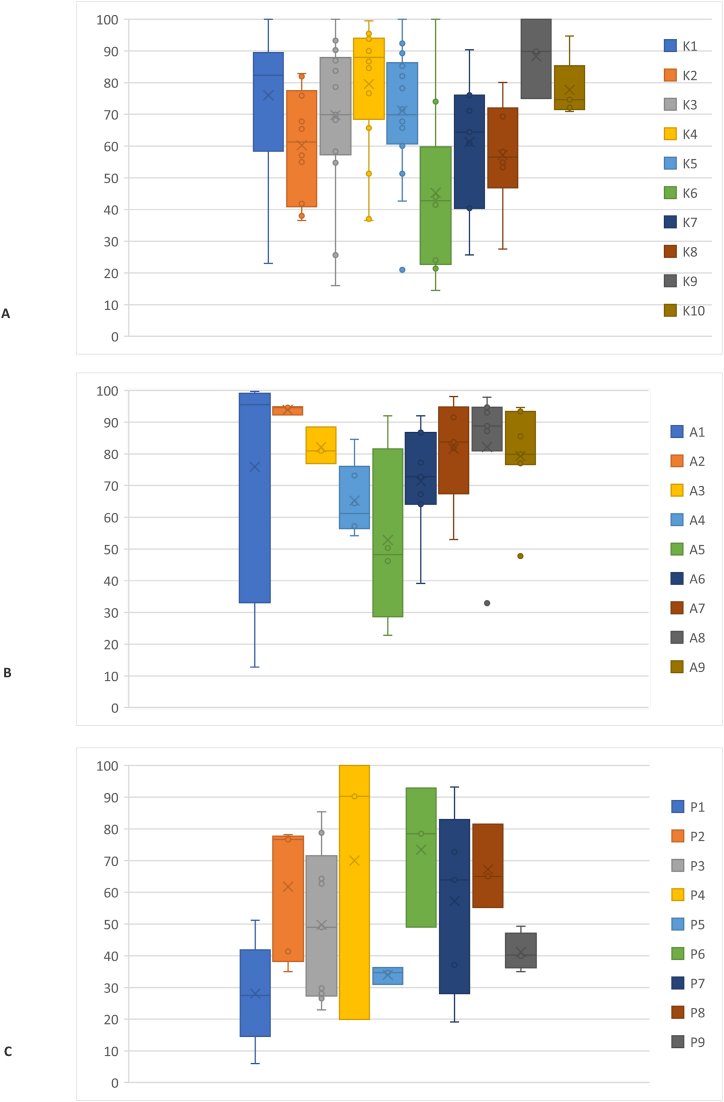
Boxplots of the prevalence of knowledge (A), attitudes (B) and practices (C) obtained in the study of prevalence of knowledge, attitudes and practices in personnel that works in restaurants and other food services.

Fig. 5 shows a summary of the study of the prevalence of knowledge, attitudes and practices of personnel working in restaurants and food services.

(A) Box plot of study prevalence of food allergy knowledge

K1. Customers with food allergies can safely consume small portions of the specific food thy are allergic to (False); K2. If an individual is having an allergic reaction, serving them water will dilute the allergen and relieve the reaction (False); K3. High heat (e.g. frying in hot oil) destroys the majority og food allergens (False); K4. A food allergic person can die from eating the food that is allergic to (True); K5. Removing the allergen from an already ready-to-serve dish makes it safe for the person allergic to it (False); K6. Lactose intolerance and milk allergy are the same thing (False); K7. The most effective management for a severe food allergy reaction is administering epinephrine (True); K8. Modern medicine can cure food allergies. (False); K9. If the buffet in a help-yourself service contains allergens, to keep it clean is enough to make it a safe option for food allergic customers (False); K10. A food allergic reaction can occur if a client touches a food item that contains the allergens, he/she is allergic to. (True).

(B) Box plot of study prevalence of food allergy attitude:

A1. Kitchen staff should be aware of food allergies; A2. Restaurants should try to satisfy special requests made by customers with food allergies; A3. Do you think that food allergies are a serious issue worth consideration?; A4. I believe I can handle correctly an emergency food allergy situation at my workplace; A5. Have you ever thought how to prevent food allergy reactions among your customers?; A6. Do you think that you are responsible for the presence of food allergens in your allergic customer served foods?; A7. It is customers’ responsibility to express their food allergies needs; A8. I know that I can provide a safe meal to clients that inform their special needs; A9. Would you like to receive further education on food allergies?

(C) Box plot of study prevalence of food allergy practices:

P1. Do you have a plan to provide safe meals to allergic clients?; P2. Would you modify a recipe for food allergy customers who request it?; P3. On your menu, do you highlight allergenic ingredients or insert a warning note to inform the presence of specific allergens like peanuts or others?; P4. I wash my hands with soap and water and change my gloves before processing allergen-free foods; P5. I post information about food allergies in the restaurant's website; P6. Sometimes I fry allergen-free foods in the same oil where we previously fried allergen containing foods; P7. Does this restaurant have a special set of utensils or equipment to prepare allergen-free food?; P8. Does this restaurant have a list of procedures with the menu recipes indicating the ingredients they are made of?; P9. Do the personnel have training focused on how to receive and deal with consumers that inform food allergies?

### 4.5 Synthesis of data by categories

The analysis of the distribution of the combined proportion of knowledge, attitude, and practices related to food allergies within the surveyed population reveals important patterns (S7 File). A distinctive pattern is observed in the distribution of knowledge about food allergies. Quintiles 4 and 5 exhibit a high level of knowledge among the population across constructs. In these categories, the combined proportions of correct responses consistently surpass the 84 % threshold. This suggests that a significant portion of the surveyed population possesses a solid understanding of food allergies and related concepts.

As for the attitude category, an interesting finding emerges. From the second quintile onwards, nearly the entire surveyed population declares a positive attitude toward the possibility of serving consumers with food allergies. The combined proportions of positive responses in this category consistently exceed the 85 % threshold. This indicates that the majority of participants display a favorable and receptive attitude towards addressing the dietary needs of consumers with food allergies.

Analyzing the distribution of the combined proportion within the practices category, a low-risk behavior is notable in specific questions (such as P4: “Do I wash my hands with soap and water and change my gloves before processing allergen-free foods?” and P7: “Does this restaurant have a special set of utensils or equipment to prepare allergen-free food?”). These questions exceed the 85 % threshold and are primarily concentrated in quintile 5. However, unlike the previous categories, the practices category shows that a significant proportion of quintiles potentially indicate higher-risk behaviors for allergic consumers. This implies that there is room for improvement in the preparation and safe management of food allergies for customers.

Both knowledge and attitudes regarding food allergies reveal strong thoughts across most quintiles of the surveyed population. However, when it comes to practices, there is a need to enhance and promote safer and more responsible behaviors in the preparation and service for consumers with food allergies, particularly in the lower quintiles.

The heterogeneity observed in the analyzed dataset reflects marked variability in the different constructs evaluated in relation to food allergies (S7 File). The inherent diversity within the data reveals significant variability in the different evaluated constructs.

In the attitudes category, represented by the construct A9: “Would you like to receive further education on food allergies?” a high heterogeneity is also present, with an I^2 index of 93,9 %. When synthesizing the results of individual studies, an accumulated proportion of 89 % is obtained for this construct.

In the realm of practices, specifically regarding the construct P3: “On your menu, do you highlight allergenic ingredients or insert a warning note to inform the presence of specific allergens like peanuts or others?” substantial heterogeneity is evident, represented by an I^2 index of 97.2 %. This indicator points to significant variability in the results of individual studies, which, when combined, result in an overall proportion of 56 %.

It is essential to note that this heterogeneity underscores the importance of interpreting the aggregated results with caution and carefully examining the contextual differences that may influence the observed outcomes in each construct. The observed variability may result from various conditions and methodological approaches among the studies, highlighting the need to consider a breadth of perspectives in the analysis of the collected information.

S7 File presents a synthesis of the prevalence analysis results for all constructs within each category: knowledge, attitudes, and practices. This includes the outcomes of meta-analyses of proportions, distribution across quintiles of the combined proportion, effect size, and heterogeneity.

## 5 Discussion

This systematic review and meta-analysis addresses a topic of growing relevance: the knowledge, attitudes, and practices of personnel serving allergic consumers in restaurants and food services.

Over the past decade, the frequency of food allergies has increased, as has the trend of dining out, emphasizing the need to understand how this issue is managed by personnel in the food industry.

The analysis of knowledge, attitudes, and practices constructs highlights areas of strength and opportunity.

Previous knowledge about food allergies and their management is fundamental in food service environments to ensure the safety of allergic customers. Although most studies show a generally acceptable level of knowledge about food allergies, specific gaps persist in critical areas such as understanding anaphylactic reactions and the proper administration of epinephrine (K7). For example, while 95 % of respondents recognize that a person with allergies can die from consuming the allergenic food (K4), only 76 % correctly identify the administration of epinephrine as the most effective measure to manage a severe allergic reaction (K7). Although there is widespread recognition of the severity of food allergies and a willingness to learn more about the topic, concerning misconceptions persist, such as the safety of consuming small portions of allergens. Troubling misconceptions about food allergies are observed, with a high percentage of participants (80 %) believing incorrect statements, such as consuming small portions of allergens being safe (K1) or 78 % believing that high heat destroys allergens (K3).

The attitude towards the responsibility of staff towards allergic customers and their willingness to continue educating themselves are encouraging aspects observed in this study. However, there is an evident gap between these intentions and actual practices, as only a minority of establishments have a plan for safe meals for allergic customers, and information about allergens on menus is scarce. Although staff largely recognize (85 %) the severity of food allergies and their responsibility towards allergic customers (A3), 84 % believe that this positive attitude does not always translate into a high level of knowledge (A4). Even when clear gaps are evident in some areas evaluated [25], this highlights the importance of aligning attitudes with specific competencies. The high frequency with which participants acknowledge being willing to receive more training on food allergies suggests a certain degree of uncertainty in managing these situations [[26], [27], [28], [29], [30], [31], [32]]. For example, although the majority of food service staff (94 %) believe they should accommodate special requests from customers with food allergies (A2), only 68 % feel confident in handling a food allergy-related emergency at their workplace (A4). Overall, staff show willingness to acquire more knowledge, with several studies reporting a positive attitude of staff towards allergic customers, and the majority of the studied population feeling capable of providing allergen-free food safely when requested by allergic customers [18,27,33].

It is relevant to highlight the shared responsibility of consumers and service providers when it comes to safely dining out [14,34]. Studies reveal that interviewed staff believe allergic individuals should communicate their condition [27,28,33,35,36]. The willingness of food service staff to receive more training on food allergies is evident (A9), suggesting a recognition of the importance of improving knowledge and skills in this area. However, this contrasts with the lack of information provided on menus, websites, and other consumer communications [28,37,38], despite menus being considered the primary source of information for allergic customers. It is essential to emphasize the importance of ongoing training for staff on issues related to food allergies and the need for effective communication between staff and allergic consumers (A7). Effective communication can foster the trust of allergic individuals in certain restaurants and services, potentially avoiding legal actions and harm [18]. This includes training on how to properly receive and handle consumers reporting food allergies (P9). Although previous studies advocated for comprehensive training for all food-handling staff [39,40], employees now express interest in receiving more focused training on food allergies. The nature of this training also deserves definition, as some participants express a preference for shorter, more specific training periods, focused on tools and behaviors to handle special situations [28]. A self-guided and structured program for employees could be a valuable option for improving safety regarding food allergies in restaurants and other food services [40].

Regarding observed practices, a lack of preventive measures to avoid allergic reactions (P1) is highlighted, along with a lack of awareness about the importance of cross-contamination with food allergens (P4). Preventing the consequences of food allergies (FA) presents a challenge, as food service staff in general do not implement preventive measures and show resistance to doing so [26,35,41,42](43). Although food service staff are usually well-informed about programs related to microbiological safety and hygiene, they often lack understanding about the importance of cross-contamination when it comes to food allergens [34]. Despite positive attitudes, there is a significant gap between intentions and practices. Only a small portion of restaurants (28 %) have a plan for safe meals for allergic customers (P1). Often, information about allergens on menus is lacking (only 56 % highlight allergens) (P3). Practices such as reusing oil for allergenic and non-allergenic foods are concerning (66 % admitted to this practice) (P6). Staff training on handling food allergies is limited, with only 41 % reporting having received training on addressing immediate needs (P9). Although most staff report having specialized utensils and equipment for handling allergen-free preparations (P7), there is still room for improvement in implementing preventive measures and communicating crucial information about food allergies in food service environments. Similarly, none of the reviewed articles report explicit plans or programs expressing an interest in providing safe food to allergic individuals [25,27,31,35,36]. This represents a significant gap because, although waiters and restaurant service staff may express their willingness to accommodate special requests, their ability to respond appropriately in emergency situations is clearly limited by the infrastructure and facilities available at work and the lack of clear instructions on what to do [17] in specific emergency situations (P1). Only two-thirds of respondents claim to have specialized utensils and equipment for handling allergen-free preparations (P7). Best preparation practices should include specific and separate references in local protocols regarding labeling, packaging, and designated locations for storing allergen-free foods and ingredients [41].

Among the limitations of this study, it must be mentioned that the diversity in methodology and geographic contexts of the analyzed studies could affect comparisons. Despite our synthesis efforts, these variations may have impacted the overall conclusions. Furthermore, when considering these limitations, the analysis of construct heterogeneity in the synthesis reveals a wide variability in results due to differences in design, population, intervention, and outcome measures. Therefore, the effect size of each study should be interpreted cautiously, and it should be acknowledged that findings may not apply universally. Heterogeneity was lower than expected in the meta-analyses of three constructs. This might be due to the limited number of studies or limited precision in estimating heterogeneity. It's important to note that while significant heterogeneity was identified in most constructs, our primary focus was summarizing information in tables rather than evaluating it within each of them.

## 6 Conclusions

In conclusion, it is evident that the implementation of specific measures in the gastronomy industry aimed at the safety of allergic consumers is an imperative step. However, these measures cannot prevail in isolation; it is essential to accompany them with educational programs and comprehensive training for customer service staff. Although more developed regions such as the US and Europe have regulations that consider food allergens, these primarily focus on packaged and labeled foods, leaving less room for prepared foods in the gastronomy industry.

In this regard, the systematic review underscores the importance of adopting effective policy and regulatory solutions in the gastronomy industry, targeting both allergic consumers and staff. The implementation of additional regulations and public policies is recommended to strengthen current evidence-based initiatives, focusing on training and establishing robust protocols.

In this context, adopting the recommendations of the Codex Alimentarius in public policies could create a more coherent and global approach to addressing food allergies in restaurants. This collaborative effort among public health, the restaurant industry, and food sector employees ultimately aims to transform establishments into safe spaces for individuals with food allergies, ensuring their well-being and peace of mind at all times.

## Funding

There was no additional funding or grant from funding agencies in the public, commercial, or not-for-profit sectors.

## Data availability

Data will be made available on request.

## CRediT authorship contribution statement

**Ximena A. Figueroa-Gómez:** Writing – review & editing, Writing – original draft, Visualization, Methodology, Investigation, Formal analysis, Conceptualization. **María Jesús Oliveras-López:** Methodology, Investigation, Data curation. **Marcelo F. Poyanco-Bugueño:** Visualization, Software, Formal analysis, Data curation. **Francisco M. Ocaña-Peinado:** Software, Formal analysis. **Herminia López-García de la Serrana:** Validation, Supervision. **Magdalena Araya Quezada:** Writing – review & editing, Writing – original draft, Validation, Project administration, Methodology, Conceptualization.

## Declaration of competing interest

The authors declare that they have no known competing financial interests or personal relationships that could have appeared to influence the work reported in this paper.
