# Supplementary material for: Knowledge, attitude, and practices of restaurant and foodservice personnel in food allergy. A systematic review and meta-analysis
Source: Heliyon. 2024 Jun 24;10(13):e33431. doi: 10.1016/j.heliyon.2024.e33431 (PMC11260967; doi:10.1016/j.heliyon.2024.e33431)
Supplement: Multimedia component 3 [file mmc3.docx]

**Supplementary File 7: Summary table of meta-analyses for proportions by construct and heterogeneity**

| **ID** | **Constructs** | **Sample size** | **No. of studies** | **Combined proportion** | **Q^*^** | **95% CI** | **effect size** | **ES^**^** | **P-value** | **I^2** |
| --- | --- | --- | --- | --- | --- | --- | --- | --- | --- | --- |
|  | **Knowledge** |  | | | | | | | | |
| K1 | Customers with food allergies can safely consume small portions of the specific food thy are allergic to (False) | 3815 | 19 | 0,80 | Q3 | 0,77 - 0,82 | 0,78 | 315,192 | 1,000 | 94,29 % |
| K2 | If an individual is having an allergic reaction, serving them water will dilute the allergen and relieve the reaction (False) | 1411 | 10 | 0,64 | Q2 | 0,61 - 0,67 | 0,62 | 72,801 | 1,000 | 87,64 % |
| K3 | High heat (e.g. frying in hot oil) destroys the majority og food allergens (False) | 3002 | 17 | 0,78 | Q3 | 0,75 - 0,81 | 0,76 | 327,314 | 1,000 | 95,11 % |
| K4 | A food allergic person can die from eating the food that is allergic to (True) | 3844 | 16 | 0,95 | Q5 | 0,92 - 0,98 | 0,95 | 425,075 | 1,000 | 96,47 % |
| K5 | Removing the allergen from an already ready-to-serve dish makes it safe for the person allergic to it (False) | 3481 | 18 | 0,84 | Q4 | 0,81 - 0,87 | 0,82 | 361,798 | 1,000 | 95,30 % |
| K6 | Lactose intolerance and milk allergy are the same thing (False) | 1209 | 9 | 0,47 | Q1 | 0,44 - 0,50 | 0,43 | 70,883 | 1,000 | 88,71 % |
| K7 | The most effective management for a severe food allergy reaction is administering epinephrine (True) | 1251 | 7 | 0,76 | Q2 | 0,72 - 0,80 | 0,74 | 137,076 | 1,000 | 95,62 % |
| K8 | Modern medicine can cure food allergies. (False) | 1215 | 6 | 0,58 | Q1 | 0,55 - 0,62 | 0,56 | 81,328 | 1,000 | 93,85 % |
| K9 | If a buffet counter (help yourself) contains allergens, but is kept clean, it may be a safe option for a food allergic customer (False). | 343 | 3 | 0,87 | Q5 | 0,78 - 0,96 | 0,87 | 8,874 | 0,988 | 77,46 % |
| K10 | A food allergic reaction can occur if a client touches a food item that contains the allergens he/she is allergic to. (True) | 961 | 5 | 0,86 | Q4 | 0,80 - 0,91 | 0,85 | 74,558 | 1,000 | 94,64 % |
| **ID** | **Attitude** |  | | | | | | | | |
| A1 | Kitchen staff should be aware of food allergies. | 1153 | 4 | 0,99 | Q5 | 0,94 - 01,05 | 0,99 | 112,136 | 1,000 | 97,32 % |
| A2 | Restaurants should try to satisfy special requests made by customers with food allergies | 1147 | 3 | 0,94 | Q4 | 0,89 - 01,00 | 0,94 | 1,478 | 0,522 | -35,35 % |
| A3 | Do you think that food allergies are a serious issue worth consideration? | 410 | 3 | 0,85 | Q2 | 0,77 - 0,93 | 0,85 | 6,173 | 0,954 | 67,60 % |
| A4 | I believe I can handle correctly an emergency food allergy situation at my workplace | 1752 | 6 | 0,68 | Q1 | 0,65 - 0,71 | 0,67 | 52,959 | 1,000 | 90,56 % |
| A5 | Have you ever thought how to prevent food allergy reactions among your customers? | 1068 | 4 | 0,85 | Q2 | 0,80 - 0,90 | 0,82 | 252,065 | 1,000 | 98,81 % |
| A6 | Do you think that you are responsible for the presence of food allergens in your allergic customer served foods? | 1726 | 7 | 0,84 | Q1 | 0,80 - 0,88 | 0,84 | 134,651 | 1,000 | 95,54 % |
| A7 | It is customers’ responsibility to express their food allergies needs. | 959 | 5 | 0,94 | Q4 | 0,88 - 01,00 | 0,94 | 118,534 | 1,000 | 96,63 % |
| A8 | I know that I can provide a safe meal to clients that inform their special needs. | 1101 | 7 | 0,94 | Q4 | 0,89 - 01,00 | 0,94 | 77,232 | 1,000 | 92,23 % |
| A9 | Would you like to receive further education on food allergies? | 1703 | 7 | 0,89 | Q3 | 0,85 - 0,94 | 0,89 | 97,081 | 1,000 | 93,82 % |
| **ID** | **Practice** |  | | | | | | | | |
| P1 | Do you have a plan to provide safe meals to allergic clients? | 666 | 5 | 0,28 | Q1 | 0,26 - 0,30 | 0,24 | 16,205 | 0,997 | 75,316 % |
| P2 | Would you modify a recipe for food allergy customers who request it? | 828 | 5 | 0,71 | Q4 | 0,67 - 0,76 | 0,70 | 51,423 | 1,000 | 92,221% |
| P3 | On your menu, do you highlight allergenic ingredients or insert a warning note to inform the presence of specific allergens like peanuts or others? | 2215 | 9 | 0,56 | Q2 | 0,54 - 0,59 | 0,51 | 283,976 | 1,000 | 97,183 % |
| P4 | I wash my hands with soap and water and change my gloves before processing allergen-free foods | 821 | 3 | 0,87 | Q5 | 0,81 - 0,93 | 0,85 | 110,156 | 1,000 | 98,184 % |
| P5 | I post information about food allergies in the restaurant's website | 643 | 3 | 0,34 | Q1 | 0,32 - 0,37 | 0,34 | 0,573 | 0,249 | -249,340 % |
| P6 | Sometimes I fry allergen-free foods in the same oil where we previously fried allergen containing foods | 663 | 3 | 0,66 | Q3 | 0,61 - 0,71 | 0,64 | 66,383 | 1,000 | 96,987 % |
| P7 | Does this restaurant have a special set of utensils or equipment to prepare allergen-free food? | 1307 | 5 | 0,86 | Q5 | 0,81 - 0,90 | 0,82 | 299,192 | 1,000 | 98,663 % |
| P8 | Does this restaurant have a list of procedures with the menu recipes indicating the ingredients they are made of? | 547 | 3 | 0,69 | Q4 | 0,63 - 0,75 | 0,68 | 24,673 | 1,000 | 91,894 % |
| P9 | Do the personnel have training focused on how to receive and deal with consumers that inform food allergies? | 1044 | 4 | 0,41 | Q2 | 0,38 - 0,43 | 0,40 | 1,703 | 0,364 | -76,157 % |

*Q=Distribution of the combined proportion by quintiles; **ES=Effect Size
