# Supplementary material for: Knowledge, attitude, and practices of restaurant and foodservice personnel in food allergy. A systematic review and meta-analysis
Source: Heliyon. 2024 Jun 24;10(13):e33431. doi: 10.1016/j.heliyon.2024.e33431 (PMC11260967; doi:10.1016/j.heliyon.2024.e33431)
Supplement: Multimedia component 4 [file mmc4.docx]

**Supplementary file 6: Constructs knowledge, attitude and practices**

**Construct: knowledge**

[K1. Customers with food allergies can safely consume small portions of the specific food thy are allergic to (False) 2](#_Toc144146792)

[K2. If an individual is having an allergic reaction, serving them water will dilute the allergen and relieve the reaction (False) 3](#_Toc144146793)

[K3. High heat (e.g. frying in hot oil) destroys the majority og food allergens (False) 4](#_Toc144146794)

[K4. A food allergic person can die from eating the food that is allergic to (True) 5](#_Toc144146795)

[K5. Removing the allergen from an already ready-to-serve dish makes it safe for the person allergic to it (False). 6](#_Toc144146796)

[K6. Lactose intolerance and milk allergy are the same thing (False) 7](#_Toc144146797)

[K7. The most effective management for a severe food allergy reaction is administering epinephrine (True) 8](#_Toc144146798)

[K8. Modern medicine can cure food allergies. (False) 9](#_Toc144146799)

[K9. If the buffet in a help-yourself service contains allergens, to keep it clean is enough to make it a safe option for food allergic customers (False). 10](#_Toc144146800)

[K10. A food allergic reaction can occur if a client touches a food item that contains the allergens he/she is allergic to. (True). 11](#_Toc144146801)

Construct: Attitud

[A1. Kitchen staff should be aware of food allergies. 12](#_Toc144146802)

[A2. Restaurants should try to satisfy special requests made by customers with food allergies. 13](#_Toc144146803)

[A3. Do you think that food allergies are a serious issue worth consideration? 14](#_Toc144146804)

[A4. I believe I can handle correctly an emergency food allergy situation at my workplace 15](#_Toc144146805)

[A5. Have you ever thought how to prevent food allergy reactions among your customers? 16](#_Toc144146806)

[A6. Do you think that you are responsible for the presence of food allergens in your allergic customer served foods? 17](#_Toc144146807)

[A7. It is customers’ responsibility to express their food allergies needs. 18](#_Toc144146808)

[A8. I know that I can provide a safe meal to clients that inform their special needs. 19](#_Toc144146809)

[A9. Would you like to receive further education on food allergies? 20](#_Toc144146810)

Construct: practices

[P1. Do you have a plan to provide safe meals to allergic clients? 21](#_Toc144146811)

[P2. Would you modify a recipe for food allergy customers who request it? 22](#_Toc144146812)

[P3. On your menu, do you highlight allergenic ingredients or insert a warning note to inform the presence of specific allergens like peanuts or others? 23](#_Toc144146813)

[P4. I wash my hands with soap and water and change my gloves before processing allergen-free foods 24](#_Toc144146814)

[P5. I post information about food allergies in the restaurant's website 25](#_Toc144146815)

[P6. Sometimes I fry allergen-free foods in the same oil where we previously fried allergen containing foods 26](#_Toc144146816)

[P7. Does this restaurant have a list of procedures with the menu recipes indicating the ingredients they are made of? 27](#_Toc144146817)

[P8. Does this restaurant have a list of procedures with the menu recipes indicating the ingredients they are made of? 28](#_Toc144146818)

[P9. Do the personnel have training focused on how to receive and deal with consumers that inform food allergies? 29](#_Toc144146819)

1. **Customers with food allergies can safely consume small portions of the specific food thy are allergic to (False)**
2. **If an individual is having an allergic reaction, serving them water will dilute the allergen and relieve the reaction (False)**
3. **High heat (e.g. frying in hot oil) destroys the majority og food allergens (False)**
4. **A food allergic person can die from eating the food that is allergic to (True)**
5. **Removing the allergen from an already ready-to-serve dish makes it safe for the person allergic to it (False).**
6. **Lactose intolerance and milk allergy are the same thing (False))**
7. **The most effective management for a severe food allergy reaction is administering epinephrine (True)**
8. **Modern medicine can cure food allergies. (False)**
9. **If the buffet in a help-yourself service contains allergens, to keep it clean is enough to make it a safe option for food allergic customers (False).**
10. **A food allergic reaction can occur if a client touches a food item that contains the allergens he/she is allergic to. (True).**
11. **Kitchen staff should be aware of food allergies.**
12. **Restaurants should try to satisfy special requests made by customers with food allergies.**
13. **Do you think that food allergies are a serious issue worth consideration?**
14. **I believe I can handle correctly an emergency food allergy situation at my workplace**
15. **Have you ever thought how to prevent food allergy reactions among your customers?**
16. **Do you think that you are responsible for the presence of food allergens in your allergic customer served foods?**
17. **It is customers’ responsibility to express their food allergies needs.**
18. **I know that I can provide a safe meal to clients that inform their special needs.**
19. **Would you like to receive further education on food allergies?**
20. **Do you have a plan to provide safe meals to allergic clients?**
21. **Would you modify a recipe for food allergy customers who request it?**
22. **On your menu, do you highlight allergenic ingredients or insert a warning note to inform the presence of specific allergens like peanuts or others?**
23. **I wash my hands with soap and water and change my gloves before processing allergen-free foods**
24. **I post information about food allergies in the restaurant's website**
25. **Sometimes I fry allergen-free foods in the same oil where we previously fried allergen containing foods**
26. **Does this restaurant have a list of procedures with the menu recipes indicating the ingredients they are made of?**
27. **Does this restaurant have a list of procedures with the menu recipes indicating the ingredients they are made of?**
28. **Do the personnel have training focused on how to receive and deal with consumers that inform food allergies?**

**Knowledge constructs contributed by each selected study**

| **Author** | **References** | **Country** | **Sample size (n)** | **Knowledge constructs** | **Right answers (n)** |
| --- | --- | --- | --- | --- | --- |
| Bailey et al.  (2011) | Restaurant staff's knowledge of anaphylaxis and dietary care of people with allergies | United Kingdom | 90 | K1: Customers with food allergies can safely consume small portions of the specific food thy are allergic to (False) | 21 |
|  |  |  |  | K2: If an individual is having an allergic reaction, serving them water will dilute the allergen and relieve the reaction (False) | 34 |
|  |  |  |  | K3: High heat (e.g. frying in hot oil) destroys the majority og food allergens (False) | 14 |
|  |  |  |  | K4: A food allergic person can die from eating the food that is allergic to (True) | 79 |
|  |  |  |  | K5: Removing the allergen from an already ready-to-serve dish makes it safe for the person allergic to it (False). | 19 |
| Wojtyniak et al.  (2013) | Knowledge of food allergy among staff of Warsaw restaurants | Poland | 78 | K4: A food allergic person can die from eating the food that is allergic to (True) | 29 |
| Bailey et al.  (2014) | Food allergy training event for restaurant staff; A pilot evaluation | United Kingdom | 11 | K1: Customers with food allergies can safely consume small portions of the specific food thy are allergic to (False) | 11 |
|  |  |  |  | K2: If an individual is having an allergic reaction, serving them water will dilute the allergen and relieve the reaction (False) | 9 |
|  |  |  |  | K3: High heat (e.g. frying in hot oil) destroys the majority og food allergens (False) | 11 |
|  |  |  |  | K5: Removing the allergen from an already ready-to-serve dish makes it safe for the person allergic to it (False). | 11 |
|  |  |  |  | K6: Lactose intolerance and milk allergy are the same thing (False) | 11 |
|  |  |  |  | K9: If the buffet in a help-yourself service contains allergens, to keep it clean is enough to make it a safe option for food allergic customers (False). | 10 |
| Wham and Sharma (2014) | Knowledge of café and restaurant managers to provide a safe meal to food allergic consumers | New Zealand | 124 | K1: Customers with food allergies can safely consume small portions of the specific food thy are allergic to (False) | 107 |
|  |  |  |  | K2: If an individual is having an allergic reaction, serving them water will dilute the allergen and relieve the reaction (False) | 52 |
|  |  |  |  | K3: High heat (e.g. frying in hot oil) destroys the majority og food allergens (False) | 109 |
|  |  |  |  | K5: Removing the allergen from an already ready-to-serve dish makes it safe for the person allergic to it (False). | 97 |
|  |  |  |  | K9: If the buffet in a help-yourself service contains allergens, to keep it clean is enough to make it a safe option for food allergic customers (False). | 93 |
| Sogut et al.  (2015) | Food allergy knowledge and attitude of restaurant personnel in Turkey | Turkey | 351 | K1: Customers with food allergies can safely consume small portions of the specific food thy are allergic to (False) | 162 |
|  |  |  |  | K2: If an individual is having an allergic reaction, serving them water will dilute the allergen and relieve the reaction (False) | 191 |
|  |  |  |  | K4: A food allergic person can die from eating the food that is allergic to (True) | 228 |
|  |  |  |  | K5: Removing the allergen from an already ready-to-serve dish makes it safe for the person allergic to it (False). | 228 |
| Shafie and Azman  (2015) | Assessment of knowledge, attitude and practice of food allergies among food handlers in the state of Penang, Malaysia. | Malaysia | 117 | K1: Customers with food allergies can safely consume small portions of the specific food thy are allergic to (False) | 58 |
|  |  |  |  | K4: A food allergic person can die from eating the food that is allergic to (True) | 60 |
|  |  |  |  | K6: Lactose intolerance and milk allergy are the same thing (False) | 17 |
| Lee and Xu  (2015) | Food Allergy Knowledge, Attitudes, and Preparedness Among Restaurant Managerial Staff | USA | 110 | K1: Customers with food allergies can safely consume small portions of the specific food thy are allergic to (False) | 90 |
|  |  |  |  | K3: High heat (e.g. frying in hot oil) destroys the majority og food allergens (False) | 75 |
|  |  |  |  | K4: A food allergic person can die from eating the food that is allergic to (True) | 99 |
|  |  |  |  | K5: Removing the allergen from an already ready-to-serve dish makes it safe for the person allergic to it (False). | 67 |
|  |  |  |  | K6: Lactose intolerance and milk allergy are the same thing (False) | 50 |
|  |  |  |  | K7: The most effective management for a severe food allergy reaction is administering epinephrine (True) | 67 |
|  |  |  |  | K8: Modern medicine can cure food allergies. (False) | 64 |
|  |  |  |  | K10: A food allergic reaction can occur if a client touches a food item that contains the allergens he/she is allergic to. (True). | 78 |
| Radke et al.  (2016) | Food allergy knowledge and attitudes of restaurant managers and staff: An EHS-Net study | USA | 644 | K1: Customers with food allergies can safely consume small portions of the specific food thy are allergic to (False) | 506 |
|  |  |  |  | K4: A food allergic person can die from eating the food that is allergic to (True) | 615 |
|  |  |  |  | K5: Removing the allergen from an already ready-to-serve dish makes it safe for the person allergic to it (False). | 595 |
| Lee and Sozen (2016) | Food allergy knowledge and training among restaurant employees | USA | 229 | K1: Customers with food allergies can safely consume small portions of the specific food thy are allergic to (False) | 205 |
|  |  |  |  | K3: High heat (e.g. frying in hot oil) destroys the majority og food allergens (False) | 160 |
|  |  |  |  | K4: A food allergic person can die from eating the food that is allergic to (True) | 219 |
|  |  |  |  | K5: Removing the allergen from an already ready-to-serve dish makes it safe for the person allergic to it (False). | 164 |
|  |  |  |  | K6: Lactose intolerance and milk allergy are the same thing (False) | 95 |
|  |  |  |  | K7: The most effective management for a severe food allergy reaction is administering epinephrine (True) | 174 |
|  |  |  |  | K8: Modern medicine can cure food allergies. (False) | 122 |
|  |  |  |  | K10: A food allergic reaction can occur if a client touches a food item that contains the allergens he/she is allergic to. (True). | 165 |
| Dupuis et al. (2016) | Food allergy management among restaurant workers in a large U.S. city | USA | 180 | K1: Customers with food allergies can safely consume small portions of the specific food thy are allergic to (False) | 159 |
|  |  |  | 174 | K2: If an individual is having an allergic reaction, serving them water will dilute the allergen and relieve the reaction (False) | 132 |
|  |  |  | 179 | K3: High heat (e.g. frying in hot oil) destroys the majority og food allergens (False) | 167 |
|  |  |  | 187 | K4: A food allergic person can die from eating the food that is allergic to (True) | 176 |
|  |  |  | 177 | K5: Removing the allergen from an already ready-to-serve dish makes it safe for the person allergic to it (False). | 158 |
| Lessa et al.  (2016) | Food Allergy Knowledge, Attitudes and Practices: A Pilot Study of the General Public and Food Handlers | Spain | 80 | K1: Customers with food allergies can safely consume small portions of the specific food thy are allergic to (False) | 66 |
|  |  |  |  | K2: If an individual is having an allergic reaction, serving them water will dilute the allergen and relieve the reaction (False) | 66 |
|  |  |  |  | K3: High heat (e.g. frying in hot oil) destroys the majority og food allergens (False) | 72 |
|  |  |  |  | K5: Removing the allergen from an already ready-to-serve dish makes it safe for the person allergic to it (False). | 34 |
| Radke et al.  (2017) | Restaurant food allergy practices - six selected sites, United States, 2014 | USA |  |  |  |
| Wen and Kwon  (2017) | Restaurant servers' risk perceptions and risk communication-related behaviors when serving customers with food allergies in the U. S | USA | 316 | K3: High heat (e.g. frying in hot oil) destroys the majority og food allergens (False) | 185 |
|  |  |  |  | K4: A food allergic person can die from eating the food that is allergic to (True) | 274 |
|  |  |  |  | K5: Removing the allergen from an already ready-to-serve dish makes it safe for the person allergic to it (False). | 162 |
|  |  |  |  | K7: The most effective management for a severe food allergy reaction is administering epinephrine (True) | 37 |
|  |  |  |  | K8: Modern medicine can cure food allergies. (False) | 87 |
| Lee and Barker  (2017) | Comparison of Food Allergy Policies and Training between Alabama (AL) and National Restaurant Industry | USA | 75 | K1: Customers with food allergies can safely consume small portions of the specific food thy are allergic to (False) | 70 |
|  |  |  |  | K3: High heat (e.g. frying in hot oil) destroys the majority og food allergens (False) | 66 |
|  |  |  |  | K4: A food allergic person can die from eating the food that is allergic to (True) | 66 |
|  |  |  |  | K5: Removing the allergen from an already ready-to-serve dish makes it safe for the person allergic to it (False). | 64 |
|  |  |  |  | K6: Lactose intolerance and milk allergy are the same thing (False) | 33 |
|  |  |  |  | K7: The most effective management for a severe food allergy reaction is administering epinephrine (True) | 48 |
|  |  |  |  | K 8: Modern medicine can cure food allergies. (False) | 52 |
|  |  |  |  | K10: A food allergic reaction can occur if a client touches a food item that contains the allergens he/she is allergic to. (True). | 57 |
| Lee and Sozen (2018) | Who knows more about food allergies - restaurant managerial staff or employees? | USA | 339 | K1: Customers with food allergies can safely consume small portions of the specific food thy are allergic to (False) | 280 |
|  |  |  |  | K3: High heat (e.g. frying in hot oil) destroys the majority og food allergens (False) | 235 |
|  |  |  |  | K4: A food allergic person can die from eating the food that is allergic to (True) | 318 |
|  |  |  |  | K5: Removing the allergen from an already ready-to-serve dish makes it safe for the person allergic to it (False). | 231 |
|  |  |  |  | K6: Lactose intolerance and milk allergy are the same thing (False) | 145 |
|  |  |  |  | K7: The most effective management for a severe food allergy reaction is administering epinephrine (True) | 241 |
|  |  |  |  | K8: Modern medicine can cure food allergies. (False) | 186 |
|  |  |  |  | K10: A food allergic reaction can occur if a client touches a food item that contains the allergens he/she is allergic to. (True). | 253 |
| McAdams et al.  (2018) | Food allergy knowledge, attitudes, and resources of restaurant employees | Canada | 208 | K1: Customers with food allergies can safely consume small portions of the specific food thy are allergic to (False) | 162 |
|  |  |  |  | K4: A food allergic person can die from eating the food that is allergic to (True) | 207 |
|  |  |  |  | K5: Removing the allergen from an already ready-to-serve dish makes it safe for the person allergic to it (False). | 194 |
|  |  |  |  | K7: The most effective management for a severe food allergy reaction is administering epinephrine (True) | 188 |
|  |  |  |  | K9: If the buffet in a help-yourself service contains allergens, to keep it clean is enough to make it a safe option for food allergic customers (False). | 187 |
|  |  |  |  | K10: A food allergic reaction can occur if a client touches a food item that contains the allergens he/she is allergic to. (True). | 197 |
| Soon  (2018) | ‘No nuts please': Food allergen management in takeaways | United Kingdom | 28 | K1: Customers with food allergies can safely consume small portions of the specific food thy are allergic to (False) | 27 |
|  |  |  |  | K2: If an individual is having an allergic reaction, serving them water will dilute the allergen and relieve the reaction (False) | 16 |
|  |  |  |  | K3: High heat (e.g. frying in hot oil) destroys the majority og food allergens (False) | 22 |
|  |  |  |  | K5: Removing the allergen from an already ready-to-serve dish makes it safe for the person allergic to it (False). | 23 |
|  |  |  |  | K6: Lactose intolerance and milk allergy are the same thing (False) | 6 |
| Jianu and Golet  (2019) | Food Allergies: Knowledge and Practice among Food Service Workers Operating in Western Romania | Romania | 121 | K1: Customers with food allergies can safely consume small portions of the specific food thy are allergic to (False) | 90 |
|  |  |  |  | K2: If an individual is having an allergic reaction, serving them water will dilute the allergen and relieve the reaction (False) | 82 |
|  |  |  |  | K3: High heat (e.g. frying in hot oil) destroys the majority og food allergens (False) | 31 |
|  |  |  |  | K5: Removing the allergen from an already ready-to-serve dish makes it safe for the person allergic to it (False). | 82 |
| Loerbroks et al.  (2019) | Food allergy knowledge, attitudes and their determinants among restaurant staff: A cross-sectional study | Germany | 295 | K1: Customers with food allergies can safely consume small portions of the specific food thy are allergic to (False) | 243 |
|  |  |  |  | K2: If an individual is having an allergic reaction, serving them water will dilute the allergen and relieve the reaction (False) | 193 |
|  |  |  |  | K3: High heat (e.g. frying in hot oil) destroys the majority og food allergens (False) | 247 |
|  |  |  |  | K4: A food allergic person can die from eating the food that is allergic to (True) | 266 |
|  |  |  |  | K5: Removing the allergen from an already ready-to-serve dish makes it safe for the person allergic to it (False). | 244 |
| Bujaka and Riekstina-Dolge  (2019) | Food allergy knowledge and practice of restaurant staff | Latvia | 154 | K1: Customers with food allergies can safely consume small portions of the specific food thy are allergic to (False) | 90 |
|  |  |  |  | K4: A food allergic person can die from eating the food that is allergic to (True) | 118 |
|  |  |  |  | K6: Lactose intolerance and milk allergy are the same thing (False) | 37 |
| Pádua et al. (2020) | Impact of a web-based program to improve food allergy management in schools and restaurants | Portugal | 146 | K1: Customers with food allergies can safely consume small portions of the specific food thy are allergic to (False) | 146 |
|  |  |  |  | K3: High heat (e.g. frying in hot oil) destroys the majority og food allergens (False) | 85 |
|  |  |  |  | K5: Removing the allergen from an already ready-to-serve dish makes it safe for the person allergic to it (False). | 88 |
|  |  |  |  | K6: Lactose intolerance and milk allergy are the same thing (False) | 108 |
|  |  |  |  | K7: The most effective management for a severe food allergy reaction is administering epinephrine (True) | 59 |
|  |  |  |  | K8: Modern medicine can cure food allergies. (False) | 55 |
| Nasseredine et al.  (2021) | Food allergy knowledge, attitudes and practices of foodservice workers at restaurants in Lebanon: Findings from a national cross-sectional study | Lebanon | 137 | K2: If an individual is having an allergic reaction, serving them water will dilute the allergen and relieve the reaction (False) | 50 |
|  |  |  |  | K3: High heat (e.g. frying in hot oil) destroys the majority og food allergens (False) | 75 |
|  |  |  |  | K4: A food allergic person can die from eating the food that is allergic to (True) | 50 |
|  |  |  |  | K5: Removing the allergen from an already ready-to-serve dish makes it safe for the person allergic to it (False). | 63 |
| Eren et al. (2021) | Food allergy knowledge, attitude, and practices of chefs in resort hotels in Turkey | Turkey | 514 | K1: Customers with food allergies can safely consume small portions of the specific food thy are allergic to (False) | 266 |
|  |  |  |  | K3: High heat (e.g. frying in hot oil) destroys the majority og food allergens (False) | 289 |
|  |  |  |  | K4: A food allergic person can die from eating the food that is allergic to (True) | 435 |

**Attitude constructs contributed by each selected study**

| **Author** | **References** | **Country** | **Sample size (n)** | **Attitude constructs** | **Right answers (n)** |  |
| --- | --- | --- | --- | --- | --- | --- |
| Bailey et al.  (2011) | Restaurant staff's knowledge of anaphylaxis and dietary care of people with allergies | United Kingdom | 90 | A4: I believe I can handle correctly an emergency food allergy situation at my workplace | 58 |  |
|  |  |  |  | A8: I know that I can provide a safe meal to clients that inform their special needs. | 73 |  |
|  |  |  |  | A9: Would you like to receive further education on food allergies? | 43 |  |
| Wojtyniak et al.  (2013) | Knowledge of food allergy among staff of Warsaw restaurants | Poland |  |  |  |  |
| Bailey et al.  (2014) | Food allergy training event for restaurant staff; A pilot evaluation | United Kingdom |  |  |  |  |
| Wham and Sharma  (2014) | Knowledge of café and restaurant managers to provide a safe meal to food allergic consumers | New Zealand | 124 | A8: I know that I can provide a safe meal to clients that inform their special needs. | 115 |  |
|  |  |  |  | A9: Would you like to receive further education on food allergies? | 95 |  |
| Sogut et al.  (2015) | Food allergy knowledge and attitude of restaurant personnel in Turkey | Turkey | 351 | A9: Would you like to receive further education on food allergies? | 269 |  |
| Shafie and Azman  (2015) | Assessment of knowledge, attitude and practice of food allergies among food handlers in the state of Penang, Malaysia. | Malaysia | 117 | A1: Kitchen staff should be aware of food allergies. | 15 |  |
|  |  |  |  | A3: Do you think that food allergies are a serious issue worth consideration? | 90 |  |
|  |  |  |  | A4: I believe I can handle correctly an emergency food allergy situation at my workplace | 99 |  |
|  |  |  |  | A5: Have you ever thought how to prevent food allergy reactions among your customers? | 54 |  |
|  |  |  |  | A6: Do you think that you are responsible for the presence of food allergens in your allergic customer served foods? | 75 |  |
|  |  |  |  | A7: It is customers’ responsibility to express their food allergies needs. | 98 |  |
|  |  |  |  | A8: I know that I can provide a safe meal to clients that inform their special needs. | 97 |  |
| Lee and Xu  (2015) | Food Allergy Knowledge, Attitudes, and Preparedness Among Restaurant Managerial Staff | USA | 110 | A3: Do you think that food allergies are a serious issue worth consideration? | 89 | |
|  |  |  |  | A6: Do you think that you are responsible for the presence of food allergens in your allergic customer served foods? | 43 | |
|  |  |  |  | A7: It is customers’ responsibility to express their food allergies needs. | 90 | |
| Radke et al.  (2016) | Food allergy knowledge and attitudes of restaurant managers and staff: An EHS-Net study | USA | 644 | A1: Kitchen staff should be aware of food allergies. | 642 |  |
|  |  |  |  | A2: Restaurants should try to satisfy special requests made by customers with food allergies. | 611 |  |
|  |  |  |  | A4: I believe I can handle correctly an emergency food allergy situation at my workplace | 471 |  |
| Lee and Sozen (2016) | Food allergy knowledge and training among restaurant employees | USA | 229 | A7: It is customers’ responsibility to express their food allergies needs. | 121 |  |
|  |  |  |  | A9: Would you like to receive further education on food allergies? | 183 |  |
| Dupuis et al. (2016) | Food allergy management among restaurant workers in a large U.S. city | USA | 183 | A3: Do you think that food allergies are a serious issue worth consideration? | 162 |  |
|  |  |  | 179 | A4: I believe I can handle correctly an emergency food allergy situation at my workplace | 97 |  |
|  |  |  | 253 | A6: Do you think that you are responsible for the presence of food allergens in your allergic customer served foods? | 184 |  |
|  |  |  | 187 | A8: I know that I can provide a safe meal to clients that inform their special needs. | 183 |  |
|  |  |  | 187 | A9: Would you like to receive further education on food allergies? | 177 |  |
| Lessa et al.  (2016) | Food Allergy Knowledge, Attitudes and Practices: A Pilot Study of the General Public and Food Handlers | Spain | 80 | A8: I know that I can provide a safe meal to clients that inform their special needs. | 26 |  |
| Radke et al.  (2017) | Restaurant food allergy practices - six selected sites, United States, 2014 | USA |  |  |  |  |
| Wen and Kwon (2017) | Restaurant servers' risk perceptions and risk communication-related behaviors when serving customers with food allergies in the U. S | USA |  |  |  |  |
| Lee and Barker (2017) | Comparison of Food Allergy Policies and Training between Alabama (AL) and National Restaurant Industry | USA |  |  |  |  |
| Lee and Sozen (2018) | Who knows more about food allergies - restaurant managerial staff or employees? | USA |  |  |  |  |
| Mc Adams et al.  (2018) | Food allergy knowledge, attitudes, and resources of restaurant employees | Canada | 208 | A2: Restaurants should try to satisfy special requests made by customers with food allergies. | 192 |  |
|  |  |  |  | A4: I believe I can handle correctly an emergency food allergy situation at my workplace | 119 |  |
|  |  |  |  | A7: It is customers’ responsibility to express their food allergies needs. | 204 |  |
|  |  |  |  | A8: I know that I can provide a safe meal to clients that inform their special needs. | 197 |  |
|  |  |  |  | A9: Would you like to receive further education on food allergies? | 178 |  |
| Soon  (2018) | ‘No nuts please': Food allergen management in takeaways | United Kingdom |  |  |  |  |
| Jianu and Golet  (2019) | Food Allergies: Knowledge and Practice among Food Service Workers Operating in Western Romania | Romania | 121 | A5: Have you ever thought how to prevent food allergy reactions among your customers? | 73 |  |
|  |  |  |  | A6: Do you think that you are responsible for the presence of food allergens in your allergic customer served foods? | 105 |  |
| Loerbroks et al.  (2019) | Food allergy knowledge, attitudes and their determinants among restaurant staff: A cross-sectional study | Germany | 295 | A1: Kitchen staff should be aware of food allergies. | 287 |  |
|  |  |  |  | A2: Restaurants should try to satisfy special requests made by customers with food allergies. | 279 |  |
|  |  |  |  | A6: Do you think that you are responsible for the presence of food allergens in your allergic customer served foods? | 197 |  |
|  |  |  |  | A7: It is customers’ responsibility to express their food allergies needs. | 270 |  |
|  |  |  |  | A8: I know that I can provide a safe meal to clients that inform their special needs. | 262 |  |
| Bujaka and Riekstina-Dolge  (2019) | Food allergy knowledge and practice of restaurant staff | Latvia |  |  |  |  |
| Pádua et al. (2020) | Impact of a web-based program to improve food allergy management in schools and restaurants | Portugal |  |  |  |  |
| Nasseredine et al.  (2021) | Food allergy knowledge, attitudes and practices of foodservice workers at restaurants in Lebanon: Findings from a national cross-sectional study | Lebanon | 137 | A1: Kitchen staff should be aware of food allergies. | 91 |  |
| Eren et al. (2021) | Food allergy knowledge, attitude, and practices of chefs in resort hotels in Turkey | Turkey | 514 | A4: I believe I can handle correctly an emergency food allergy situation at my workplace | 298 |  |
|  |  |  |  | A5: Have you ever thought how to prevent food allergy reactions among your customers? | 473 |  |
|  |  |  |  | A6: Do you think that you are responsible for the presence of food allergens in your allergic customer served foods? | 473 |  |
|  |  |  |  | A9: Would you like to receive further education on food allergies? | 480 |  |

**Practices constructs contributed by each selected study**

| **Author** | **References** | **Country** | **Sample size (n)** | **Practices constructs** | **Right answers (n)** |
| --- | --- | --- | --- | --- | --- |
| Bailey et al.  (2011) | Restaurant staff's knowledge of anaphylaxis and dietary care of people with allergies | United Kingdom |  |  |  |
| Wojtyniak et al.  (2013) | Knowledge of food allergy among staff of Warsaw restaurants | Poland |  |  |  |
| Bailey et al.  (2014) | Food allergy training event for restaurant staff; A pilot evaluation | United Kingdom |  |  |  |
| Wham and Sharma  (2014) | Knowledge of café and restaurant managers to provide a safe meal to food allergic consumers | New Zealand | 124 | P1: Do you have a plan to provide safe meals to allergic clients? | 7 |
|  |  |  |  | P8: Does this restaurant have a list of procedures with the menu recipes indicating the ingredients they are made of? | 81 |
| Sogut et al.  (2015) | Food allergy knowledge and attitude of restaurant personnel in Turkey | Turkey |  |  |  |
| Shafie and Azman (2015) | Assessment of knowledge, attitude and practice of food allergies among food handlers in the state of Penang, Malaysia. | Malaysia | 117 | P1: Do you have a plan to provide safe meals to allergic clients? | 38 |
|  |  |  |  | P3: On your menu, do you highlight allergenic ingredients or insert a warning note to inform the presence of specific allergens like peanuts or others? | 31 |
|  |  |  |  | P 9: Do the personnel have training focused on how to receive and deal with consumers that inform food allergies? | 41 |
| Lee and Xu  (2015) | Food Allergy Knowledge, Attitudes, and Preparedness Among Restaurant Managerial Staff | USA | 110 | P2: Would you modify a recipe for food allergy customers who request it? | 85 |
| Radke et al.  (2016) | Food allergy knowledge and attitudes of restaurant managers and staff: An EHS-Net study | USA |  |  |  |
| Lee and Sozen (2016) | Food allergy knowledge and training among restaurant employees | USA | 229 | P2: Would you modify a recipe for food allergy customers who request it? | 175 |
|  |  |  |  | P3: On your menu, do you highlight allergenic ingredients or insert a warning note to inform the presence of specific allergens like peanuts or others? | 97 |
|  |  |  |  | P5: I post information about food allergies in the restaurant’s website | 71 |
| Dupuis et al. (2016) | Food allergy management among restaurant workers in a large U.S. city | USA | 186 | P4: I wash my hands with soap and water and change my gloves before processing allergen-free foods | 37 |
|  |  |  |  | P7: Does this restaurant have a list of procedures with the menu recipes indicating the ingredients they are made of? | 69 |
| Lessa et al.  (2016) | Food Allergy Knowledge, Attitudes and Practices: A Pilot Study of the General Public and Food Handlers | Spain | 80 | P1: Do you have a plan to provide safe meals to allergic clients? | 41 |
| Radke et al.  (2017) | Restaurant food allergy practices - six selected sites, United States, 2014 | USA | 644 | P3: On your menu, do you highlight allergenic ingredients or insert a warning note to inform the presence of specific allergens like peanuts or others? | 192 |
|  |  |  | 277 | P7: Does this restaurant have a list of procedures with the menu recipes indicating the ingredients they are made of? | 53 |
|  |  |  | 277 | P8: Does this restaurant have a list of procedures with the menu recipes indicating the ingredients they are made of? | 153 |
|  |  |  | 644 | P9: Do the personnel have training focused on how to receive and deal with consumers that inform food allergies? | 261 |
| Wen and Kwon (2017) | Restaurant servers' risk perceptions and risk communication-related behaviors when serving customers with food allergies in the U. S | USA | 144 | P3: On your menu, do you highlight allergenic ingredients or insert a warning note to inform the presence of specific allergens like peanuts or others? | 123 |
| Lee and Barker (2017) | Comparison of Food Allergy Policies and Training between Alabama (AL) and National Restaurant Industry | USA | 110 | P2: Would you modify a recipe for food allergy customers who request it? | 86 |
|  |  |  | 185 | P3: On your menu, do you highlight allergenic ingredients or insert a warning note to inform the presence of specific allergens like peanuts or others? | 69 |
|  |  |  | 75 | P5: I post information about food allergies in the restaurant’s website | 26 |
|  |  |  | 75 | P9: Do the personnel have training focused on how to receive and deal with consumers that inform food allergies? | 37 |
| Lee and Sozen (2018) | Who knows more about food allergies - restaurant managerial staff or employees? | USA | 339 | P2: Would you modify a recipe for food allergy customers who request it? | 260 |
|  |  |  |  | P3: On your menu, do you highlight allergenic ingredients or insert a warning note to inform the presence of specific allergens like peanuts or others? | 166 |
|  |  |  |  | P5: I post information about food allergies in the restaurant’s website | 123 |
| McAdams et al.  (2018) | Food allergy knowledge, attitudes, and resources of restaurant employees | Canada | 208 | P1: Do you have a plan to provide safe meals to allergic clients? | 48 |
|  |  |  |  | P3: On your menu, do you highlight allergenic ingredients or insert a warning note to inform the presence of specific allergens like peanuts or others? | 164 |
|  |  |  |  | P7: Does this restaurant have a list of procedures with the menu recipes indicating the ingredients they are made of? | 133 |
|  |  |  |  | P9: Do the personnel have training focused on how to receive and deal with consumers that inform food allergies? | 83 |
| Soon  (2018) | ‘No nuts please': Food allergen management in takeaways | United Kingdom | 28 | P6: Sometimes I fry allergen-free foods in the same oil where we previously fried allergen containing foods | 26 |
| Jianu and Golet  (2019) | Food Allergies: Knowledge and Practice among Food Service Workers Operating in Western Romania | Romania | 121 | P4: I wash my hands with soap and water and change my gloves before processing allergen-free foods | 121 |
|  |  |  |  | P6: Sometimes I fry allergen-free foods in the same oil where we previously fried allergen containing foods | 95 |
|  |  |  |  | P7: Does this restaurant have a list of procedures with the menu recipes indicating the ingredients they are made of? | 88 |
| Loerbroks et al.  (2019) | Food allergy knowledge, attitudes and their determinants among restaurant staff: A cross-sectional study | Germany | 295 | P3: On your menu, do you highlight allergenic ingredients or insert a warning note to inform the presence of specific allergens like peanuts or others? | 83 |
| Bujaka and Riekstina-Dolge  (2019) | Food allergy knowledge and practice of restaurant staff | Latvia | 154 | P3: On your menu, do you highlight allergenic ingredients or insert a warning note to inform the presence of specific allergens like peanuts or others? | 35 |
| Pádua et al. (2020) | Impact of a web-based program to improve food allergy management in schools and restaurants | Portugal | 146 | P8: Does this restaurant have a list of procedures with the menu recipes indicating the ingredients they are made of? | 119 |
| Nasseredine et al.  (2021) | Food allergy knowledge, attitudes and practices of foodservice workers at restaurants in Lebanon: Findings from a national cross-sectional study | Lebanon | 137 | P1: Do you have a plan to provide safe meals to allergic clients? | 11 |
|  |  |  | 40 | P2: Would you modify a recipe for food allergy customers who request it? | 14 |
| Eren et al. (2021) | Food allergy knowledge, attitude, and practices of chefs in resort hotels in Turkey | Turkey | 514 | P4: I wash my hands with soap and water and change my gloves before processing allergen-free foods | 464 |
|  |  |  |  | P6: Sometimes I fry allergen-free foods in the same oil where we previously fried allergen containing foods | 252 |
|  |  |  |  | P7: Does this restaurant have a list of procedures with the menu recipes indicating the ingredients they are made of? | 479 |

|  | Study that did not contribute to this category |
| --- | --- |
